# Consistent explainable image quality assessment for medical imaging

**DOI:** 10.1007/s13755-025-00411-0

**Published:** 2026-01-20

**Authors:** Caner Ozer, Arda Guler, Aysel Turkvatan Cansever, Ilkay Oksuz

**Affiliations:** 1 Computer Engineering Department, Istanbul Technical University, Istanbul, 34467 Türkiye; 2 Mehmet Akif Ersoy Thoracic and Cardiovascular Surgery Training Research Hospital, Istanbul, Türkiye; 3 Department of Applied Mathematics, University of Twente, Enschede, 7522 the Netherlands; 4 School of Biomedical Engineering and Imaging Sciences, King’s College London, London, SE1 7EH UK

**Keywords:** Image quality assessment, Interpretability, Consistency, Foreign object detection, LVOT detection

## Abstract

**Purpose:**

Medical image quality assessment is crucial, as poor-quality images can lead to misdiagnosis. Manual quality labeling is tedious for large studies and may produce misleading results. While automated analysis of image quality has been studied, little focus has been given to explaining and quantifying methodologies. This study proposes an explainable image quality assessment system, validated in two contexts: foreign object detection in Chest X-Rays (Object-CXR) and Left Ventricular Outflow Tract (LVOT) detection in Cardiac MRI.

**Methods:**

Our explainable pipeline employs NormGrad, an algorithm that efficiently localizes image quality issues by comparing the classifier’s saliency maps against several baseline saliency detectors. Additionally, a novel metric, the Difference of Means (DoM), is introduced to assess the consistency of saliency detectors across different network architectures.

**Results:**

We compare NormGrad with a range of saliency detection methods and demonstrate its superior performance in measuring the faithfulness of the saliency detectors. Specifically, NormGrad achieved a repeated Pointing Game score of 0.863 for Object-CXR and 0.778 for LVOT datasets, significantly outperforming other saliency detectors. Furthermore, our explainable pipeline shows strong consistency, with DoM scores of 0.001 for Object-CXR and 0.005 for LVOT datasets, indicating high reliability across different reproduced models. The code and experiments are publicly available at https://github.com/canerozer/explainable-iqa.

**Conclusion:**

The proposed system, powered by NormGrad, significantly improves the reliability of automated medical image quality evaluations. The introduction of the Difference of Means metric offers a unique way to assess saliency detector consistency, supporting NormGrad’s potential for widespread clinical adoption.

## Introduction

Maintaining high image quality during a medical scan is essential for obtaining a clear diagnosis of a patient. However, some distortions in the image quality may hinder an accurate analysis of the acquired medical scan, and this could eventually lead to inaccurate diagnostic conclusions by the physician. The reasons include physiological and patient-based motion artefacts in Magnetic Resonance Imaging (MRI) [1, 2], mistriggering-based motion artefacts in MRI, which happen due to incorrect ECG triggering during the MRI scan [3], or the contrast problem in CT due to low-dose imaging [4]. Assuming the existence of a reference image, it is possible to evaluate the level of distortion through image quality assessment metrics, such as Peak Signal-to-Noise Ratio (PSNR), Structural Similarity Index [5], or among more recent methods: HaarPSI [6] and DISTS [7]. However, often such a reference does not exist and manual labelling based on qualitative analysis is required [8]. This procedure is generally tedious and prone to human error.

In many clinical scenarios, obtaining a reference-quality scan is infeasible, making no-reference image quality assessment (NR-IQA) essential [9]. Localized quality degradations are especially challenging, as small but diagnostically critical regions can compromise the entire scan. Common examples include foreign objects such as clips or buttons obscuring the lung or cardiac areas in Chest X-Ray images [10], and the unintended appearance of the Left Ventricular Outflow Tract (LVOT) in long-axis cardiac MRI due to suboptimal planning [11]. While recent work has advanced global multi-class IQA and domain adaptation, local IQA problems require models that can reliably identify and reason about specific regions where quality issues arise. Automatic IQA systems remain crucial for filtering low-quality data in large-scale studies, improving acquisition pipelines, and ensuring trustworthy performance in downstream tasks such as segmentation and diagnosis.

Explainability has become a crucial aspect of evaluating the trustworthiness of automatic diagnostic systems in particularly in black-box deep learning applications [12]. Given that a deep classifier provides only the predicted label for a given medical scan, it is imperative to find cues that can help ensure the decision has been made based on relatable sets of features. Additionally, it has recently been shown that physicians, lacking task-specific knowledge, can benefit from the recommendations of explainable AI more than human advice, particularly in the form of visual annotations [13]. However, an explainability analysis is not well-studied for understanding the deep learning models for medical image quality assessment, even though this research field has recently been challenged in Arun et al. [14] and Jin et al. [15]. Also, the methodology responsible for the explainability analysis should quantitatively analyze the precision and consistency aspects of the saliency maps [16]. All these aspects become vital, especially in the presence of abnormalities in the medical scan in terms of medical image quality.

The quantitative evaluation measure should be reasonable for understanding the effect of assisting radiologists with saliency maps in a clinical environment. Despite several available measures, such as insertion/deletion score [17], overlap ratio, or even structural similarity [14] between the saliency map and ground truth annotations, we consider the Pointing Game [18] to be the most practical metric for clinical interpretability applications for the following reasons.

First, insertion and deletion procedures may generate out-of-distribution images during masking or deblurring and primarily consider the ranking of saliency values rather than their actual magnitudes, limiting their interpretability in a clinical setting [19, 20]. Second, overlap-based metrics, such as Dice score or intersection-over-union between a thresholded saliency map and a ground-truth bounding box, can produce misleading segmentation masks that do not faithfully reflect the model’s attention, making them potentially unreliable for evaluation. Third, ground truth annotations do not preserve luminance or contrast information, so using structural similarity may not accurately assess the quality of the generated saliency maps. Finally, radiologists focus on specific regions when performing diagnoses, and while these gazes are associated with generated saliency maps [21], the Pointing Game encapsulates this information by measuring the proximity between the ground truth and the peak location of the saliency map. Accordingly, we base our interpretability analysis on the Pointing Game while ensuring that the experiments are clinically plausible.

In this paper, we propose a consistent, explainable image quality assessment pipeline based on NormGrad [22]. This paper has been built upon our previous work, [23], in which we proposed using NormGrad to localise the regions that cause image quality degradation on Chest X-Rays by pointing out foreign objects. Here, we extend this approach to off-axis 4-chamber Cardiac MRI scans by localizing the LVOT regions and demonstrate the strength of NormGrad by stress-testing a number of saliency detectors. To the best of our knowledge, this is the first approach that uses NormGrad for medical image *quality* assessment and the first paper to perform a comprehensive, explainable image quality assessment on Chest X-Ray and Cardiac MRI data. The main contributions of our approach are listed as follows:

- Extensive validation experiments are performed to assess the quantitative performance of NormGrad for medical image quality assessment, contrasting it with other explainability methods. We measured the effect of using smoothing on the saliency maps, randomising the neural network models, repeating the experiments from scratch a different number of times, and changing the network architecture. To make our approach clinically plausible, we used the Pointing Game as our core metric.
- The Difference of Means (DoM) metric is proposed to demonstrate the consistency of a saliency detector whenever there is a change in the network architecture.
- The pipeline is validated using Object-CXR (X-Ray) and LVOT Detection (Cardiac MRI) datasets.

The remainder of the paper is organized as follows. Section 2 provides the relevant literature for this work. Section 3 defines the mathematical background of the methods used for our explainability analysis, and Sect. 4 presents our results of qualitative and quantitative analyses to evaluate the performance of a variety of explainability methods. Further discussion is made on the methods in Sect. 5, before providing our conclusions.

## Literature review

In this section, we provide an overview of related works on image quality assessment and interpretability.

### Image quality assessment

Medical Image Quality Assessment (IQA) is becoming an emergent research field with the advent of deep learning-based approaches. Maintaining sufficient image quality is crucial for alleviating potential diagnostic errors in both automatic and manual image quality assessment procedures, as detailed in Chow et al. [9]. Predominantly, domain knowledge holds the key when assessing quality. Therefore, historically, most approaches have been applicable to a single modality and specific types of image quality assessment problems. In addition, reference images may not be available depending on the problem domain, which has led to the development of the No-Reference Image Quality Assessment (NR-IQA) field. Indeed, this has driven the creation of suitable methods and datasets for medical image analysis that have not been evaluated using reference-based quality metrics, such as PSNR and SSIM.

For instance, Sujit et al. [24] proposed a model for structural brain MRI quality assessment using a small CNN architecture to automatically measure overall image quality. Additionally, Hu et al. [25] proposed a diagnostic quality assessment dataset composed of Chest X-Rays and used a semantic segmentation-guided multi-label classification framework. Mortamet et al. [26] identified several factors contributing to quality degradation in brain MRIs and proposed two different quality measures based on voxel-wise quality assessment. Treating quality assessment as a regression problem, Abdi et al. [27] assessed the quality of echocardiography cines in five different views using a convolutional neural network with multi-head LSTM layers. Similarly, in our previous approach, Ozer et al. [23] assessed the quality of Chest X-Ray scans depending on the existence of foreign objects.

Recent advances have further expanded IQA to more generalizable and multi-class settings, including attention-guided unsupervised domain adaptation for cardiac MRI [28] and multi-teacher knowledge distillation for robust motion artifact detection [29].

In Chen et al. [30], an intravascular ultrasound dataset was constructed, consisting of three quality labels. They used a ResNet-18 model [31] with squeeze and excitation modules [32] to predict the quality label. In Bottani et al. [33], a dataset of 3D T1-weighted brain MRIs was curated, and their quality was determined based on properness, gadolinium usage, and diagnostic quality. Classification of these acquisitions was performed using 3D CNNs. Moreover, Ma et al. [1] developed a T2-weighted abdomen MR dataset consisting of good quality scans and scans with motion artifacts. They used a 4-layer CNN framework to automatically assess scan quality. Gagoski et al. [34] performed prospective medical image quality assessment on fetal brain HASTE (Half Fourier Single-shot Turbo spin-Echo) images with a teacher-student network architecture. Additionally, Oksuz et al. [2] further advanced motion artifact detection by adopting not only a synthetic motion artifact generation scheme but also by performing detection and overcoming quality issues during the reconstruction phase of short-axis cardiac MRI scans by turning it into a full-reference IQA problem.

Further, dual-domain cardiac coverage assessment [35], meta-learning for cross-cohort multi-class IQA [36], and automated Chest X-Ray quality assessment using deep learning and linear regression cascades for positioning and acquisition verification on large-scale datasets [37] have advanced the field further. Foundational prompt-based models have also been proposed for more generalizable, cross-modality NR-IQA [38].

### Interpretability

Interpretability is an important area of research at the intersection of medical image analysis and deep learning. Early approaches to interpretability in deep learning include the DeConvNet [39] approach, which visualizes intermediate-level activations by reverting them to the original image dimensionality. However, the use of gradients for *saliency map* visualizations was initiated by Grad [40], which calculates the derivative of the output with respect to the input image. Subsequently, due to the nature of the ReLU activation function and advancing network architectures, Guided Backpropagation [41] was proposed to alter the gradient backpropagation mechanism by also considering the sign values of the upstream gradient. Following this, various methods that combine activations and gradients have become more prevalent in the general computer vision and medical image analysis literature. These include Input x Grad [42], Grad-CAM [43], Guided Grad-CAM [43], and NormGrad [22]. In addition to these methods, Integrated Gradients [44], Score-CAM [45], Smooth-Grad [46], and RISE [17] have proposed to use different iterative procedures to calculate the saliency map. These methods take substantially longer time to generate the saliency map compared to methods like Input x Grad.

Beyond standard saliency detectors, model-agnostic interpretability frameworks such as SHAP [47] and LIME [48] estimate feature importance through repeated input perturbations and surrogate modeling. While flexible, these approaches are computationally intensive and rely solely on changes in model outputs, without accessing internal gradients or feature activations. In contrast, model-specific CAM-based methods such as NormGrad efficiently leverage gradients and intermediate activations to generate high-resolution, spatially coherent explanations.

Recently, the use of saliency detectors to demonstrate diagnostic findings through heatmaps has become increasingly popular in medical imaging. For instance, [49] used Grad-CAM to localise the brain regions that do and do not contribute to schizophrenia on the spatial source phase maps. [50] modified the DenseNet architecture to improve the visualization of Grad-CAM without any fall in accuracy for arrhythmia detection using ECG. [51] adopted a Vision Transformer-based architecture for COVID-19 severity quantification and used a variety of CAM and deep taylor decomposition to show the saliency maps. [52] leveraged the saliency maps to train a diabetic retinopathy grading framework in a self-supervised fashion. In this regard, we benchmark NormGrad with other saliency detection methods for the interpretable no-reference image quality assessment problem.

Even though the research community demonstrates the findings shown by a saliency method qualitatively, works that provide a systematic quantitative analysis to validate the relationship between a saliency method and the model’s performance in the domain of medical image analysis are relatively recent. For example, [53] evaluates different saliency detectors for an arrhythmia detection task on electrocardiograms, where the authors used intersection over union, Pointing Game, and the absolute difference of insertion and deletion metrics. Similarly, [54] focuses on the breast tissue classification task and applies Class Activation Mapping to construct the saliency maps. The evaluation of saliency maps is performed with the Pointing Game. Lastly, [55] generates a counterfactual saliency map for Chest X-Ray lesion detection using a conditioned generative adversarial network. The saliency map is evaluated with Energy-based Pointing Game [45] and deletion metric. In this work, we find it intuitive to adopt the Pointing Game for evaluating the saliency maps for medical image quality assessment, specifically those generated for LVOT and foreign object localisation tasks for Cardiac MRIs and Chest X-Rays, respectively. This is mainly due to the fact that the Pointing Game is a highly related concept to the gaze of physicians when they are performing a diagnosis.

## Method

In this section, we briefly describe the use of NormGrad for medical image quality assessment, aiding in constructing a saliency map of deep neural network-based classifiers. We consider two variants of NormGrad in this work. NormGrad Single computes the saliency map based on the gradients and activation maps of a single target layer, while NormGrad Multi aggregates gradients and activation maps from multiple layers to produce a *combined* saliency map.

We start by assuming that there exists a pre-trained neural network from which we intend to extract knowledge about a specific target layer $k_t$. We also designate its preceding layers as *p* and succeeding layers as *q*. Given an input image ${\textbf {x}} \in \mathbb {R}^{C \times H \times W}$, where *C* represents the number of input channels, and *H* and *W* represent the size of the image, we can define the relationship between ${\textbf {x}}^{in} \in \mathbb {R}^{K \times H' \times W'}$, ${\textbf {x}}^{out} \in \mathbb {R}^{K' \times H' \times W'}$, and the network output ${\textbf {y}}$ as follows:

1 $$\begin{aligned} \begin{aligned} {\textbf {x}}^{in}&= p({\textbf {x}}) \\ {\textbf {x}}^{out}&= k_t({\textbf {x}}^{in}) \\ {\textbf {y}}&= q({\textbf {x}}^{out}). \end{aligned} \end{aligned}$$

We define the gradient w.r.t. the parameters of layer $k_t$ as ${\textbf {g}}^{out} \in \mathrm{I\!R}^{K' \times H' \times W'}$ and the activations of the same layer as ${\textbf {x}}^{out}$. Unless stated otherwise, the gradient is calculated by assuming that ${\textbf {y}}$ is the ground-truth class label, while this may not be true for all samples.

**Fig. 1:**
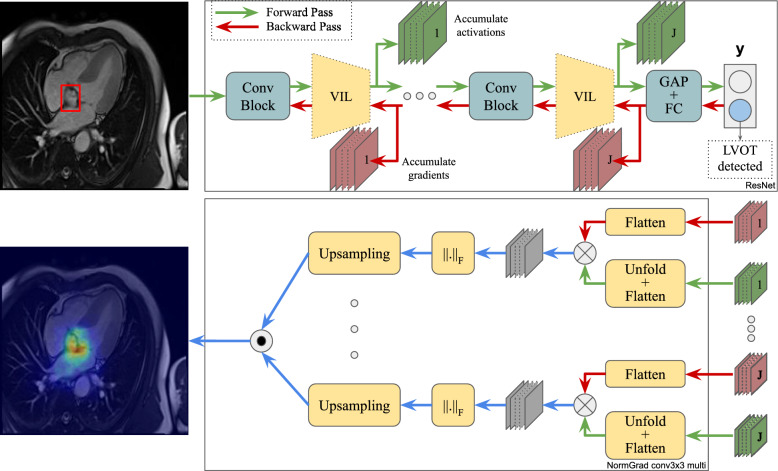
NormGrad Framework: The top part illustrates the flow of a neural network, where multiple virtual identity layers (VILs) are positioned right after the convolutional blocks to accumulate the activations and gradients from different locations. The bottom part demonstrates how these values are utilized to generate a unified heat map using NormGrad. Regions with high and low values are indicated by red and blue colors within the heat maps. "Conv Block" denotes a convolutional layer with batch normalization and ReLU activation function, while "GAP" and "FC" operations refer to Global Average Pooling and Fully-Connected layers, respectively. The input image displays the appearance of the Left Ventricular Outflow Tract in a 4-chamber Cardiac MRI scan, highlighted by a red bounding box

Additionally, consider the presence of a virtual identical layer $\tilde{k}_t$ right after the layer $k_t$, with an output denoted as $\tilde{{\textbf {x}}}^{out}$ and satisfying the property $\tilde{{\textbf {x}}}^{out} = {\textbf {x}}^{out}$. The purpose of introducing this layer is to ensure the collection of activations and gradients from the same point in the network. Moreover, this layer can be selected from scaling or convolutional layers. In this regard, we accumulate the output activations, $\tilde{{\textbf {x}}}^{out}$, and the corresponding upstream gradient, ${\textbf {g}}^{out}$, of the layer $\tilde{k}_t$.

### NormGrad

NormGrad [22] directly utilizes ${\textbf {g}}^{out}$ without needing to estimate a weight vector from either gradients or activations. It also allows the use of $\tilde{{\textbf {x}}}^{out}$, enabling the availability of both gradient and activations at a specific location within a neural network. This is facilitated by incorporating a virtual identity layer $\tilde{k_t}$ immediately after the layer of interest $k_t$. Moreover, the selection of a virtual identity layer also influences spatial contributions, and also the generated saliency map, as illustrated in Table 1.

**Table 1:** Spatial contributions, shapes and saliency map formulas of different virtual identity layer choices

Layer	Spatial contribution	Shape	Saliency map
Scaling	${\textbf {g}}^{out}_{u} \odot {\textbf {x}}^{out}_{u}$	$K'$	$\Vert {\textbf {g}}^{out} \odot {\textbf {x}}^{out} \Vert _F$
Conv $N \times N$	${\textbf {g}}^{out}_u {{\textbf {x}}^{out}_{u, N \times N}}^\intercal$	$K' \times N^2K'$	$\Vert {\textbf {g}}^{out} \Vert _F$ $\Vert {\textbf {x}}^{out}_{N \times N} \Vert _F$

The first row corresponds to a scaling layer where the spatial contribution is obtained by calculating the element-wise multiplication of the upstream gradient, ${\textbf {g}}^{out}$, with the activations, ${\textbf {x}}^{out}$. This results in a saliency map that is directly equal to the Frobenius Norm of the spatial contribution. However, for the second row, additional operations are needed to calculate the spatial contributions and the corresponding saliency maps for an $N \times N$ convolutional virtual identity layer. Suppose that the output relation of the convolution operation using the unfolded version of the input tensor, ${\textbf {X}}_{N \times N}^{out} \in \mathrm{I\!R}^{N^2K \times H'W'}$, and the reshaped version of the parameters of the layer $\tilde{k}_t$, $\tilde{{\textbf {W}}}_{N \times N} \in \mathrm{I\!R}^{K' \times N^2K}$, is expressed in the form of

2 $$\begin{aligned} \tilde{{\textbf {X}}}^{out} = \tilde{{\textbf {W}}}_{N \times N} {\textbf {X}}_{N \times N}^{out}, \end{aligned}$$

which uses matrix multiplication to express $\tilde{{\textbf {X}}}^{out} \in \mathrm{I\!R}^{K' \times H'W'}$. The unfold operation extracts $N \times N$ patches from the input tensor, ${\textbf {x}}_{out}$ in order to accelerate the convolution operation. If we denote each of the column elements of ${\textbf {X}}_{N \times N}^{out}$ as ${\textbf {x}}_{u, N \times N}^{out} \in \mathrm{I\!R}^{N^2 K'}$, we can find the spatial contribution by calculating the gradient of loss with respect to $\tilde{{\textbf {W}}}_{N \times N}$ which is

3 $$\begin{aligned} \begin{aligned} \frac{dL}{d\tilde{{\textbf {W}}}_{N \times N}}&= \sum _{u \in \Omega } \frac{d}{d\tilde{{\textbf {W}}}}_{N \times N} \langle {\textbf {g}}^{out}_u, \tilde{{\textbf {W}}}_{N \times N} {\textbf {x}}^{out}_{u, N \times N} \rangle \\ &= \sum _{u \in \Omega } {\textbf {g}}_u^{out} {{\textbf {x}}^{out}_{u, N \times N}}^\intercal . \end{aligned} \end{aligned}$$

Since the Frobenius Norm of an outer product can be decomposed into the multiplication of Frobenius Norms, we can calculate the resulting saliency map by first computing the Frobenius Norms of ${\textbf {g}}^{out}$ and ${\textbf {x}}^{out}_{N \times N}$ separately and multiplying these two matrices. The general procedure for transforming the spatial contributions into saliency maps is defined as the aggregation, which concludes by upsampling the saliency maps to the original image size, [*H*, *W*], to obtain the final output for a single-layer scenario, ${\textbf {m}}_{NG}$.

An additional feature of NormGrad is its ability to combine saliency maps from multiple layers within a neural network, generated using gradients and activations. In this paper, we define this as NormGrad Multi (denoted by the letter (M)), and we use a uniform setting for calculating the combined saliency map, which involves taking the geometric mean of the provided saliency maps. Moreover, for a fair comparison, we use identical virtual identity layers when generating the combined saliency map. This approach provides a unified and unbiased source of information about what the network has learned by integrating outputs from different layers. In other words, if we have *J* saliency maps, the combined saliency map ${\textbf {M}}_{NG}$ is obtained by:

4 $$\begin{aligned} {\textbf {M}}_{NG} = \Pi _{j=1}^J \root J \of {{\textbf {m}}_{NG}^{(j)}}, \end{aligned}$$

where ${\textbf {M}}_{NG}$ becomes the final output as the whole procedure is demonstrated in Fig. 1.

**Algorithm 1:**
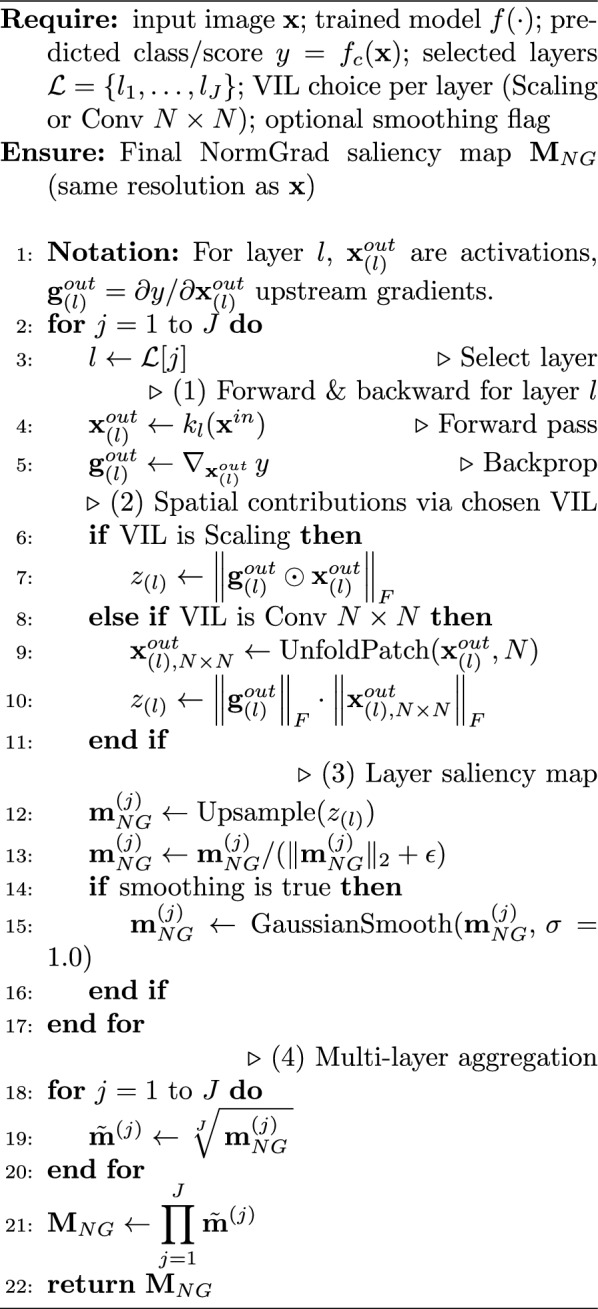
NormGrad per-layer VIL computation and multi-layer aggregation

## Experimental results

We perform our experiments on two datasets which are Object-CXR [10] and the LVOT dataset. We provide details about the datasets in Sect. 4.1, our general approach in evaluating the saliency maps in Sect. 4.2, measure the effect of smoothing the saliency maps in Sect. 4.3, make a comparison during the initialization and post-training phases in Sect. 4.4, and compare the variability across different neural network models in Sect. 4.5. We make the code and the experiments available on https://github.com/canerozer/explainable-iqa.

### Datasets

Object-CXR is a benchmarking dataset with the objective of recognizing and localizing foreign objects on Chest X-Rays. It contains a total of 10, 000 Chest X-Ray images, with 5, 000 images including foreign objects and 5, 000 images without.

Left Ventricular Outflow Tract (LVOT) detection is a cardiac MR dataset where the presence of LVOT is a local quality issue that hinders the accurate analysis of atrial regions. The dataset is composed of a range of 4-chamber cardiac MRI scans from 690 2D+ time patient records, with an even number of samples with and without LVOT. We apply a patient-wise splitting on the dataset, using 551 patients for training, 69 patients for validation, and 70 patients for testing. Since our network is designed to handle 2D images, we consider each of the slices of the four-chamber view independently. Hence, we expand the number to 8, 682 good quality and 8, 522 LVOT samples.

For the Object-CXR dataset, we directly use the bounding boxes released by JFHealthcare as part of the original benchmark, which were manually produced by expert annotators. For the LVOT dataset, ground-truth annotations were generated through a two-step process. First, an experienced cardiologist manually delineated the LVOT region using a pixel-wise segmentation mask on all slices where the structure appeared. Second, we computed the tightest axis-aligned bounding box that enclosed the segmentation mask, and used this bounding box solely for Pointing Game evaluation. A senior radiologist reviewed a randomly selected subset of the segmentation masks and confirmed their correctness.

#### Object-CXR

We train ResNet-34 [31] and EfficientNet-B0 [56] models, both predicting whether there is at least one foreign object present or not, given a resized $600 \times 600 \times 3$ image. We fine-tune these models, previously trained on the ImageNet dataset [57], for 20 epochs using a batch size of 16 and a cross-entropy loss function. We adapt our input by triplicating it on the channel axis three times and replace the last layer of the pre-trained model, which now has two output neurons, with random parameters using He initialization [58]. In order to optimise the parameters during the training procedure, we use the Stochastic Gradient Descent with Momentum optimiser, with a learning rate set to 0.005, and the learning rate reduced by a factor of 10 every five epochs. Also, to prevent overfitting during training, we use colour jittering, affine transformations, and horizontal flips as data augmentations. Finally, we keep the best-performing model, based on validation accuracy. The performance of the models in terms of AUC score and accuracy is presented in Table 7, where we observe that the peak performance was achieved in the first trial of the ResNet-34 (R34) model, reaching an accuracy of 0.870 and an AUC score of 0.938.

#### LVOT detection

We train ResNet-50 and EfficientNet-B0 models, which predict the presence or absence of LVOT, given a $224 \times 224 \times 3$ input image. Similar to the Object-CXR, we use an ImageNet pre-trained model and fine-tune the model with Stochastic Gradient Descent with Momentum optimizer, setting the learning rate to 0.0002 and weight decay to 0.0005. The fine-tuning procedure takes 60 epochs using a batch size of 64 when the cross-entropy loss function is used. Additionally, the same types of data augmentations employed in the foreign object detection task are used. Our best LVOT detection model achieves top performance in the first trial of the ResNet-50 (R50) model, with an accuracy and AUC score of 0.971 and 0.998, respectively. For the rest of the paper, we refer to this dataset as the LVOT dataset.

### Experimental evaluation on the saliency maps

In order to analyse the saliency maps, we conduct a qualitative and quantitative assessment of the validation and testing sets for the Object-CXR and LVOT datasets. We begin by examining the impact of applying smoothing to the saliency maps generated by these methods. Subsequently, we delve into randomisation experiments, evaluating the saliency maps under two conditions: (i) complete randomisation of model parameters and (ii) adoption of convolutional layer parameters from ImageNet with randomised classification layer. We compare these outcomes with the results obtained from the trained models, showcasing their repeated Pointing Game scores. Lastly, we present our reproducibility results, intended to observe the consistency in Pointing Game accuracies across different models, specifically ResNet and EfficientNet.

To highlight the abilities of NormGrad, we do not only stick to using the penultimate layer (conv4.2) of ResNet-50. We also use the spatial contribution at $J=4$ different layers, e.g., conv2.0, conv3.0, conv4.0, and conv4.2 after aggregating the saliency maps corresponding to numerous layers of the ResNet architecture. For EfficientNet models instead, despite NormGrad Single also using the penultimate layer of the model (features.8.2), we aggregate the spatial contribution at $J=10$ layers, namely, features.0.0, features.1.0, features.2.0, features.3.0, features.4.0, features.5.0, features.6.0, features.7.0, features.8.0, and, features.8.2. Then, we compare our results with other saliency detector baselines such as Grad-CAM [43], Guided Grad-CAM [43], Guided Backpropagation [41], and Input x Gradient [42].

In our study, we use Pointing Game [18] for quantitatively analysing the abilities of saliency detectors. Our purpose is to detect if saliency maps show a correspondence with the ground-truth bounding boxes given a medical scan. In Pointing Game, we measure this correspondence by finding the maximum value of a saliency map and then checking the maximum value’s proximity to the ground-truth with an offset, $\tau$. We take the default value, $\tau =15$, for both datasets. If the maximum value is close enough to the bounding boxes, we define the saliency map to be accurate. If we name the number of accurate saliency maps with *T* and inaccurate ones with *F*, we can derive an accuracy metric *A* such that:

5 $$\begin{aligned} A = \frac{T}{T + F} \end{aligned}$$

### Effect of smoothing the saliency maps

**Fig. 2:**
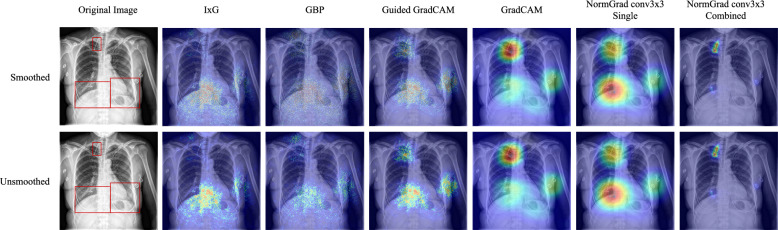
The smoothing operation when used on different interpretability methods. Although IxG (Input x Grad), GBP (Guided Backpropagation), and Guided GradCAM’s saliency outputs change heavily, we do not observe such drastic changes in the saliency maps generated by GradCAM and NormGrad. Red bounding boxes demonstrate the ground-truth annotations for foreign objects

This study investigates the effects of smoothing on the performance of various saliency detectors for medical image quality problems. Motivated by the work of [18], which suggests that some methods such as Grad x Input tend to output noisy saliency maps and require a smoothing operation to reduce sparsity and noise, a Gaussian kernel with a standard deviation of 1.0 was applied to smooth the saliency maps. The results of smoothing the saliency maps are shown in Fig. 2, where it is observed that saliency maps generated by GradCAM and NormGrad are stable, but Input x Grad (IxG), Guided Backpropagation (GBP), and Guided GradCAM are not. We also notice that the conv3x3 multi model of NormGrad successfully focuses on all target foreign objects of interest, especially on the clip at the top. Furthermore, Table 2 demonstrates that the changes in Pointing Game accuracies are insignificant for both LVOT and Object-CXR datasets when NormGrad or GradCAM is used. However, smoothing becomes increasingly crucial for methods that directly use gradient information, as seen in the first three rows of Table 2. Therefore, smoothing was applied in all comparisons to provide fairness across all saliency detectors while considering the noisiness factor of Input x Grad, Guided Backpropagation, and Guided Grad-CAM. Despite this, the success of NormGrad is still evident, while the Multi versions of NormGrad appear to be more consistent when their performance on both datasets is considered.

**Table 2:** Pointing Game accuracies for all available saliency methods, with and without the application of smoothing, are reported

	LVOT		Object-CXR	
	Unsmoothed	Smoothed	Unsmoothed	Smoothed
Input × Grad	0.000	0.098	0.162	0.670
Guided Backpropagation	0.000	0.135	0.132	0.648
Guided GradCAM	0.051	0.319	0.256	0.640
GradCAM	0.582	0.554	0.572	0.546
NormGrad Scaling	0.472	0.468	**0.850**	**0.852**
NormGrad Scaling (M)	0.627	0.626	0.840	0.838
NormGrad Conv1×1	0.431	0.430	0.848	0.848
NormGrad Conv1×1 (M)	0.639	0.639	0.840	0.838
NormGrad Conv3×3	0.295	0.292	**0.850**	0.850
NormGrad Conv3×3 (M)	**0.640**	**0.649**	0.846	0.846

Smoothing has only a slight effect on saliency detectors like NormGrad and GradCAM, while it becomes more crucial for noisier saliency detectors such as Input × Grad, Guided Backpropagation, and Guided GradCAM. The best-performing methods are underlined and highlighted in bold.

### Randomisation and repeatability experiments

In this part of our study, our goal is to demonstrate interpretability by measuring the Pointing Game accuracy of randomly initiated and trained models. We are influenced by the work of [14], which claims that saliency maps have several shortcomings, as saliency detectors are not always robust to randomisation, repeatability, and reproducibility. To measure the effect of randomisation on the saliency maps, we examined two different configurations to initialise the models. First, we use a model with all parameters randomised (Fully Randomised, FR) and second, we inherit an ImageNet [57] model for convolutional layer parameters and randomise only the final fully-connected layer (Semi Randomised, SR). Our purpose in analysing the models during the initialisation stage is to see whether it is possible to catch any significant difference in the interpretability maps after training the neural network. Hence, we assess whether we can use this information as an alternative way of measuring a deep learning model’s performance in terms of explainability. During full randomisation, we used He initialisation [58] to randomise the weight parameters whereas for semi-randomisation, we used the ImageNet pre-trained model parameters. While repeating these randomisation experiments three times to have an estimate with high confidence, we also train three models with different seeds to demonstrate the performance as a result of training. We also make a comparison between pre- and post-trained models’ performance. In all our experiments, we report the mean and standard deviation of the Pointing Game accuracy.

Higher Pointing Game scores for fully randomised models than for semi-randomised models can occasionally occur because broad architectural activation and gradient patterns may overlap with the target region by chance, relative to the expected behavior of a model that has not learned any meaningful representation. These randomised networks are included only as diagnostic baselines to help assess the stability of saliency extraction under different initialization conditions, and their scores do not correspond to meaningful localization capability.

#### Results for the LVOT experiments

**Fig. 3:**
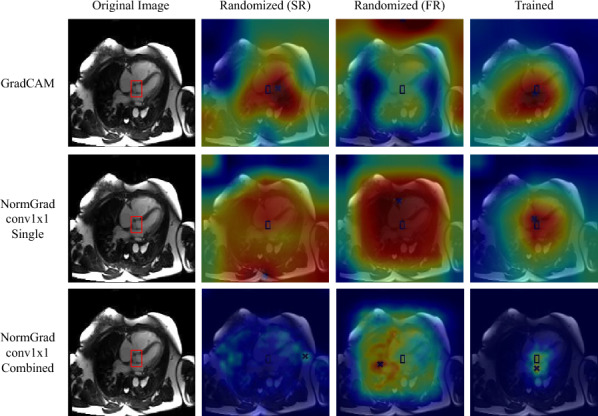
Saliency maps before and after training. Learning appropriate representations has an enormous effect on the quality of saliency maps. Still, combining the saliency map information of different layers is crucial with regard to precision. The red bounding box highlights the LVOT region as the ground-truth

In Table 3, we demonstrate the randomisation and repeatability experiments for the LVOT dataset. First, we notice a significant improvement in the Pointing Game accuracies after training the networks and running all of the saliency detection methods. As we demonstrate in Fig. 3, we associate it with the appropriately learned representations to fulfill the task and aggregate the information coming from different layers. This also leads to another statement that it is almost obligatory to train the neural networks for making them point to relevant regions of interest.

**Table 3:** Pointing Game accuracies (mean±standard deviation) of all available saliency methods when the model is initiated via ImageNet parameters except for the final fully-connected layer (Semi-randomised, SR), random parameters at full (Fully-randomised, FR), and repeatedly trained and examined (Repeated) for the **LVOT** dataset

	LVOT		
	SR	FR	Repeated
Input × Grad	0.001±0.001	0.048±0.015	0.106±0.058
Guided Backpropagation	0.004±0.002	0.103±0.041	0.152±0.075
Guided GradCAM	0.027±0.025	0.044±0.057	0.325±0.075
GradCAM	0.061±0.104	0.008±0.014	0.547±0.020
NormGrad Scaling	0.069±0.029	0.007±0.002	0.497±0.140
NormGrad Scaling (M)	0.066±0.038	0.057±0.023	0.600±0.054
NormGrad Conv1×1	0.048±0.000	0.011±0.016	0.479±0.139
NormGrad Conv1×1 (M)	0.054±0.011	0.082±0.079	**0.611**±**0.051**
NormGrad Conv3×3	0.073±0.000	0.215±0.041	0.430±0.185
NormGrad Conv3×3 (M)	0.118±0.006	0.057±0.023	0.602±0.061

Trained models illustrate superior performance over the randomised models, and NormGrad Multi configurations outweigh the baselines after training

Secondly, utilizing the multi versions of NormGrad can result in substantial improvements in Pointing Game accuracy compared to the performance of single-layer saliency detectors. Transitioning from GradCAM to the multi layer setting of NormGrad Conv1x1, we observe an enhancement in our Pointing Game accuracy from 0.547 to 0.611. Further advancements are evident upon adopting the multi layer setting in place of the single-layer setting of NormGrad. The most notable performance improvement is observed in the NormGrad Conv3x3 setting, achieving a repeated Pointing Game accuracy of 0.602 from 0.430 by utilizing multiple layers. This improvement can be attributed to the efficiency of NormGrad Multi in localizing small target regions of interest, considering the activation maps and gradients of four different layers. Lastly, we cannot draw a conclusive statement for the LVOT dataset regarding whether using pre-trained parameters would enhance the initial performance of Pointing Game accuracy since, for some NormGrad settings, fully-randomised models outperform their semi-randomised counterparts. This observation can be linked to the LVOT region’s small size compared to the image, suggesting that the multi configuration of NormGrad, utilizing gradients and activations from various layers, is crucial for addressing this limitation.

**Fig. 4:**
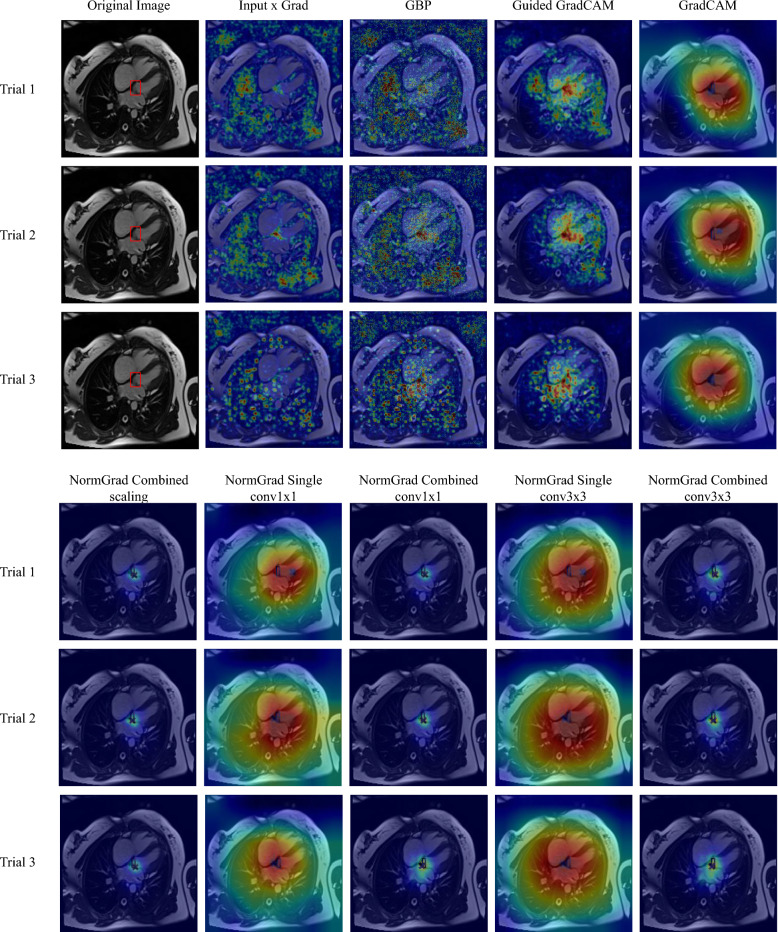
Repeatability results for a sample from the LVOT Test Set (Best viewed in zoom). Consistent results are achieved for GradCAM, Guided GradCAM, and NormGrad methods, while the precision is the best for the Multi-setting of NormGrad. The LVOT region is indicated with a red bounding box, and the most salient point is marked with a cross

The consistency of NormGrad methods after retraining the models is also visually evaluated by comparing them with the baseline methods, revealing noticeable changes in saliency maps. As shown in Fig. 4, Input x Grad and Guided Backpropagation (GBP) present issues with additional focus on the background. GradCAM may exhibit saliency outside the cardiac area or fail to focus on the target region of interest in case of misclassification, and Guided GradCAM is reliant on the performance of GradCAM and GBP. However, NormGrad maintains consistency by covering the cardiac area in saliency maps and even enhances precision by combining saliency maps generated by multiple layers.

#### Results for the object-CXR experiments

**Fig. 5:**
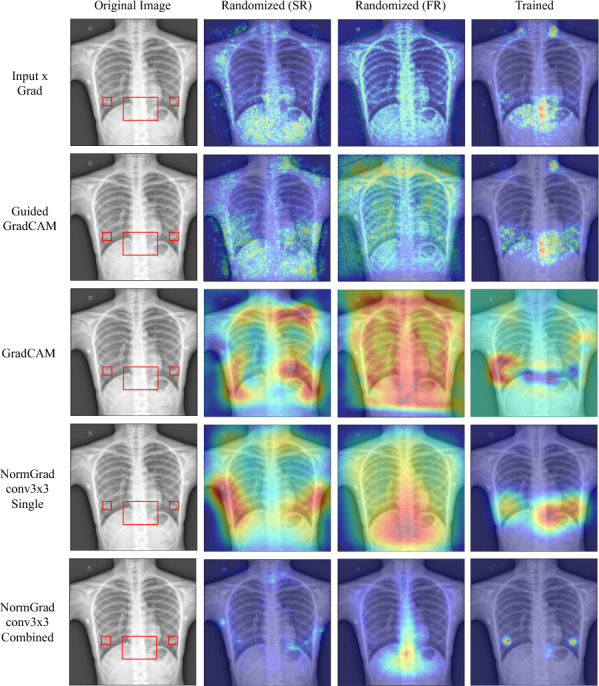
Saliency maps before and after training demonstrate the significant impact of learning appropriate representations on their quality. Red bounding boxes highlight the ground-truth annotations for foreign objects

The results for the Object-CXR dataset are presented in Table 4, indicating a significant improvement in methods after training the models, similar to the LVOT dataset. As depicted in Fig. 5, this improvement is visually apparent after training the models. However, it is important to note that not all trained representations are relevant. In the "Trained" column of Fig. 5, saliency detectors like Input x Grad and Guided GradCAM highlight a region on the left side of the neck, not present for NormGrad-based methods, as seen in the bottom two rows of the same figure. NormGrad successfully identifies all foreign objects and even improves precision when using the multi-setting of NormGrad. Moreover, the standard deviations of the repeatability experiments on the Object-CXR dataset for the NormGrad methods are relatively smaller than the baselines’ standard deviations, demonstrating the confidence and utility of the NormGrad methods as an unbiased interpretability measurement tool regardless of task repetition. There is also an insignificant difference among the NormGrad methods, all of which outperform the baseline models. Interestingly, GradCAM has the lowest repeated Pointing Game metric among all saliency detectors, raising questions about its ability to point to relevant regions through saliency maps, as illustrated in Fig. 5. In summary, NormGrad provides the best results, achieving the highest mean repeated Pointing Game performance of 0.853 using the single layer and Conv3x3 setting. Additionally, unlike the results for randomisation in the LVOT detection task, there is an improvement in the Pointing Game accuracy as a result of using ImageNet features, attributed to the increased area of the foreign objects of interest.

**Table 4:** Pointing Game accuracies (mean±standard deviation) of all available saliency methods when the model is initiated via ImageNet parameters except for the final fully-connected layer (Semi randomised, SR), random parameters at full (Fully randomised, FR), and repeatedly trained and examined (Repeated) for the **Object-CXR** dataset

	Object-CXR		
	SR	FR	Repeated
Input × Grad	0.111±0.028	0.185±0.041	0.663±0.013
Guided Backpropagation	0.091±0.008	0.160±0.007	0.640±0.053
Guided GradCAM	0.166±0.055	0.200±0.028	0.645±0.012
GradCAM	0.200±0.066	0.093±0.103	0.545±0.018
NormGrad Scaling	0.279±0.021	0.126±0.032	0.852±0.000
NormGrad Scaling (M)	0.320±0.029	0.147±0.053	0.843±0.005
NormGrad Conv1×1	0.280±0.000	0.153±0.025	0.851±0.006
NormGrad Conv1×1 (M)	0.325±0.004	0.185±0.027	0.839±0.010
NormGrad Conv3×3	0.298±0.000	0.153±0.017	**0.853**±**0.003**
NormGrad Conv3×3 (M)	0.334±0.016	0.193±0.027	0.845±0.005

Trained models exhibit significant performance over randomised models, and all NormGrad configurations outperform the baselines after training

### Reproducibility experiments

Another criterion for evaluating the reliability of saliency detectors is their reproducibility on different architectures. In this context, we assess their performance whenever we train a completely different network architecture. We compare the saliency detection performance of ResNet with EfficientNet-B0 [56] for both tasks and propose a basic metric named "Difference of Means" (DoM) to measure the consistency under a change in network architecture. From our perspective, a saliency detector should be model-agnostic, and the generated saliency map should only depend on the input image. Consequently, the Pointing Game scores corresponding to different model architectures need to be similar, and the DoM measure provides a quantification of the consistency of the saliency detection method. To calculate the DoM measure, we obtain the repeated Pointing Game metric for each of the network architectures, and we find the absolute difference between their means. A lower DoM score indicates a more consistent saliency detection method, whereas a higher DoM score signals inconsistency in the saliency detection method across different architectures.

Pointing Game accuracies form empirical sets of scalar outcomes, not samples from a continuous probability model. Distributional divergences such as Kullback–Leibler and Jensen-Shannon therefore do not apply in this regime. In contrast, Maximum Mean Discrepancy (MMD) [59] provides a nonparametric comparison suitable for empirical scalar sets. In the one-dimensional case with a linear kernel, MMD reduces to the absolute difference of the means, which corresponds exactly to the DoM metric we employ. This correspondence allows DoM to be interpreted as a principled, distribution-based measure of consistency between saliency detectors across architectures.

**Table 5:** Pointing Game performances are compared across two different network architectures: ResNet (R50) and EfficientNet (EB0)

	LVOT		
	R50	EB0	DoM
Input × Grad	0.106±0.058	0.218±0.042	0.112
Guided Backpropagation	0.152±0.075	0.318±0.049	0.166
Guided GradCAM	0.325±0.075	0.453±0.035	0.128
GradCAM	0.547±0.020	0.569±0.027	0.022
NormGrad Scaling	0.497±0.140	**0.778**±**0.020**	0.281
NormGrad Scaling (M)	0.600±0.054	0.620±0.010	0.020
NormGrad Conv1×1	0.479±0.139	0.754±0.041	0.275
NormGrad Conv1×1 (M)	**0.611**±**0.051**	0.634±0.018	0.023
NormGrad Conv3×3	0.430±0.185	0.728±0.033	0.298
NormGrad Conv3×3 (M)	0.602±0.061	0.607±0.020	**0.005**

The conv3×3 multi-layer setting of NormGrad achieves the lowest Difference of Means (DoM) score for the LVOT dataset. Given the small-scale nature of the LVOT quality issue, the multi-layer setting of NormGrad performs better and more consistently than the baseline methods

**Table 6:** Pointing Game performances are evaluated on two network architectures: ResNet (R34) and EfficientNet (EB0)

	Object-CXR		
	R34	EB0	DoM
Input × Grad	0.663±0.013	0.617±0.036	0.046
Guided Backpropagation	0.640±0.053	0.595±0.045	0.045
Guided GradCAM	0.645±0.012	0.784±0.005	0.139
GradCAM	0.545±0.020	0.771±0.005	0.226
NormGrad Scaling	0.852±0.000	0.857±0.010	0.005
NormGrad Scaling (M)	0.843±0.005	0.856±0.002	0.013
NormGrad Conv1×1	0.851±0.006	0.850±0.000	**0.001**
NormGrad Conv1×1 (M)	0.839±0.010	0.851±0.008	0.011
NormGrad Conv3×3	**0.853**±**0.003**	**0.863**±**0.002**	0.009
NormGrad Conv3×3 (M)	0.845±0.005	0.859±0.007	0.014

The conv1×1 single-layer setting of NormGrad delivers the best performance for the Object-CXR dataset. Notably, all NormGrad-based methods outperform the baselines and demonstrate greater consistency, largely due to the scale of the quality issue involved

**Fig. 6:**
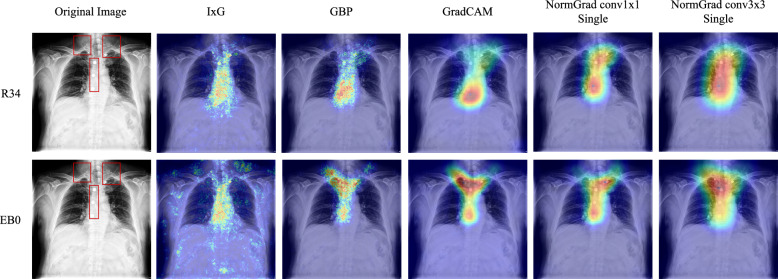
Reproducibility results for a sample from Object-CXR Test Set when ResNet-34 (R34) and EfficientNet-B0 (EB0) architectures are utilized for foreign object detection task. Consistent results are achieved for NormGrad methods since it focuses on all three foreign objects of interest. Red bounding boxes demonstrate the ground-truth annotations for foreign objects

In Tables 5 and 6, we evaluate the performance of baseline methods and NormGrad on the LVOT and Object-CXR datasets using two different architectures. First, we observe that the performance of NormGrad methods surpasses that of the baselines on both datasets, except for GradCAM when the ResNet-50 (R50) architecture is used for the LVOT detection task. Notably, when single-layer methods perform poorly, NormGrad becomes more effective and precise by combining information from different layers, as all multi-layer settings of NormGrad exceed the baselines. Second, the best performance is achieved by the single-layer version of NormGrad for the LVOT detection task using the EfficientNet-B0 (EB0) model, while there is a slight discrepancy between this setting and the best setting for the Object-CXR benchmark. However, we also notice significant differences in mean Pointing Game performance when comparing these two models under the single-layer version of NormGrad, particularly for the LVOT detection task. Although this issue does not occur for the Object-CXR benchmark, the use of multiple layers is crucial for accurate model interpretation, as indicated by the minimal DoM measure on both datasets. The differences in the saliency maps generated by different models are illustrated in Fig. 6 for the Object-CXR dataset.

It is worth mentioning the effect of using different VIL types, as their behavior varies noticeably across datasets and configurations. We observe distinct behaviors of the visualization layer (VIL) types depending on the dataset. For Object-CXR, differences among Scaling, Conv1×1, and Conv3×3 are minimal, with all variants achieving similarly high Pointing Game accuracies. Because foreign objects in Object-CXR are comparatively large and visually prominent, precise spatial modeling through the VIL plays a less critical role. For LVOT, however, the choice of VIL has a substantial impact. In the single-layer configuration, NormGrad Scaling performs best among the VIL types. In the multi-layer configuration, Conv1×1 achieves the highest performance, with differences being more pronounced in the EB0 architecture than R50. This pattern arises from the characteristics of the LVOT detection problem: in our dataset, the LVOT region tends to be localized near the image center, making global channel-wise reweighting (Scaling and Conv1×1) more effective than larger spatial kernels.

## Discussion and conclusions

In this work, we introduced an automatic and explainable framework for medical image quality assessment using NormGrad to explain its decisions by pointing to relevant regions of interest. We compared the results of NormGrad with various baseline-category saliency detectors under specific conditions, employing the Pointing Game metric. In our study, we initially presented the saliency detector performance under smoothing conditions by applying Gaussian smoothing to the generated saliency maps. Then, we evaluated the saliency detector performance under two different randomisation schemes, comparing them with their trained counterparts. Finally, we reported the saliency detector performance when different neural network architectures were employed, and proposed a basic but intuitive metric named "Difference of Means" (DoM), which measures the consistency between the saliency detection performance on two different architectures.

Comparing other saliency detection methods, we observe that smoothing had a negligible effect on NormGrad, as indicated by its minimal impact on performance for both datasets. Furthermore, the method effectively showcased the learning capability of a neural network by demonstrating a significant difference in the Pointing Game metric between the randomised and repeated versions of a neural network. It is also important to note that, using the multiple layer setting of NormGrad provides a reliable approach to interpretability from a more global perspective by integrating information from different layers. While a significant difference can be achieved by using the multi version for the LVOT detection task, this difference became insignificant for the Object-CXR task, although numerous cases still demonstrated the superiority of using the multi-setting of NormGrad in terms of precision. Ultimately, we can assert that the multi-setting of NormGrad exhibits more consistency in terms of the DoM metric and outperforms the baseline models in Pointing Game scores. Therefore, NormGrad emerges as a versatile method suitable for various medical image quality assessment tasks.

Our approach still has limitations. First, it is unlikely to use NormGrad for sensitive predictive tasks such as segmentation in radiation therapy planning [60], where the tissue may vary in size. If the tissue is sufficiently large compared to the image size, NormGrad may not accurately localize the tissue, potentially causing radiation therapy damage to healthy cells. This limitation extends to tasks with a substantial amount of data available, allowing the training of a segmentation framework with pixel-level ground truth annotations. Second, the Pointing Game metric has inherent constraints. It considers a saliency map successful if it touches any part of a bounding box, which can oversimplify evaluation in images containing many or large regions of interest. Although this is not an issue for LVOT detection, where there is a single well defined region, it is more problematic for foreign object detection, which may involve multiple targets in a single image.

Furthermore, the Pointing Game metric assumes that the quality-related target can be localized using a bounding box. This assumption does not hold for global image quality issues, such as movement-based image degradation, where the degradation affects the entire image and cannot be meaningfully annotated with discrete bounding boxes.

We also acknowledge that saliency maps can be misleading, for example when they highlight clinically irrelevant structures (e.g., objects outside the heart and thoracic area) or when the model is overconfident on borderline-quality images. Therefore, we recommend that saliency maps be treated as decision support rather than ground truth and interpreted by radiologists in conjunction with their own judgment and established image quality criteria. Finally, the application of the Difference of Means (DoM) metric in this work has been restricted to two commonly used architectures for the sake of brevity. Although these results provide initial insights, extending DoM based evaluation across a broader set of architectures would offer a more comprehensive understanding of a saliency detector performance.

We consider that our approach can be applied immediately after the image acquisition stage to promptly identify cases with quality errors. By assisting the radiologists and pointing directly to regions of interest through saliency maps, it becomes possible to make an immediate decision for re-acquisition without any human intervention. Furthermore, the verification of a saliency detector for different tasks can be conducted using our proposed experimentation setting and the DoM metric. As for future work, we plan to incorporate NormGrad saliency maps during the training stage of image quality assessment and enhancement frameworks.

## Data Availability

The LVOT dataset cannot be made publicly available due to patient privacy concerns; however, the Object-CXR dataset is available at https://academictorrents.com/details/fdc91f11d7010f7259a05403fc9d00079a09f5d5/tech.

## A classification model performance

We report the accuracy (Acc) and area under the curve (AUC) scores for the LVOT and Object-CXR datasets using the ResNet (R34, R50) and EfficientNet (EB0) architectures in Table 7. T1, T2, and T3 represent our three trials using different random seed values. For both datasets, the best performance is achieved in the first trial, T1.

**Table 7:** Accuracy (Acc) and area under curve (AUC) scores for all models. Numbers in bold and underlined font show the best performance during a trial

	LVOT				Object-CXR			
	R50		EB0		R34		EB0	
	Acc	AUC	Acc	AUC	Acc	AUC	Acc	AUC
T1	**0.971**	**0.998**	**0.929**	**0.978**	**0.870**	**0.938**	**0.858**	**0.937**
T2	0.949	0.994	0.867	0.949	0.853	**0.938**	0.857	0.936
T3	0.943	0.992	0.915	0.971	0.865	0.931	0.856	0.936
